# Exploring the Role of Lactoferrin in Managing Allergic Airway Diseases among Children: Unrevealing a Potential Breakthrough

**DOI:** 10.3390/nu16121906

**Published:** 2024-06-17

**Authors:** Alessandra Gori, Giulia Brindisi, Maria Daglia, Michele Miraglia del Giudice, Giulio Dinardo, Alessandro Di Minno, Lorenzo Drago, Cristiana Indolfi, Matteo Naso, Chiara Trincianti, Enrico Tondina, Francesco Paolo Brunese, Hammad Ullah, Attilio Varricchio, Giorgio Ciprandi, Anna Maria Zicari

**Affiliations:** 1 Department of Mother-Child, Urological Science, Sapienza University of Rome, 00161 Rome, Italy; alessandra.gori85@gmail.com (A.G.); giulia.brindisi@uniroma1.it (G.B.); 2 Department of Pharmacy, University of Napoli Federico II, Via D. Montesano 49, 80131 Naples, Italy; maria.daglia@unina.it (M.D.); alessandro.diminno@unina.it (A.D.M.); hammadrph@gmail.com (H.U.); 3 International Research Center for Food Nutrition and Safety, Jiangsu University, Zhenjiang 212013, China; 4 Department of Woman, Child and General and Specialized Surgery, University of Campania “Luigi Vanvitelli”, 80138 Naples, Italy; michele.miragliadelgiudice@unicampania.it (M.M.d.G.); dinardogiulio@gmail.com (G.D.); cristianaind@hotmail.com (C.I.); 5 CEINGE-Biotecnologie Avanzate, Via Gaetano Salvatore 486, 80145 Naples, Italy; 6 Laboratory of Clinical Microbiology & Microbiome, Department of Biomedical Sciences for Health, University of Milan, 20122 Milan, Italy; lorenzo.drago@unimi.it; 7 UOC Laboratory of Clinical Medicine, MultiLab Department, IRCCS Multimedica, 20138 Milan, Italy; 8 Allergy Center, IRCCS Istituto Giannina Gaslini, 16147 Genoa, Italy; matteo.naso1992@gmail.com (M.N.); chiaratrincianti@gaslini.org (C.T.); 9 Pediatric Clinic, Fondazione IRCCS Policlinico San Matteo, 27100 Pavia, Italy; enrico.tondina01@universitadipavia.it; 10 Primary Care Paediatrics, ASL Caserta, 81100 Caserta, Italy; francescopaolobrunese@gmail.com; 11 Department of Otolaryngology, University of Molise, 86100 Campobasso, Italy; attilio.varricchio@aivas.it; 12 Allergy Clinic, Casa di Cura Villa Montallegro, 16145 Genoa, Italy; gio.cip@libero.it

**Keywords:** atopic diseases, allergic rhinitis, asthma, anti-inflammatory, antioxidant, immunomodulation, lactoferrin

## Abstract

The prevalence of allergic diseases has dramatically increased among children in recent decades. These conditions significantly impact the quality of life of allergic children and their families. Lactoferrin, a multifunctional glycoprotein found in various biological fluids, is emerging as a promising immunomodulatory agent that can potentially alleviate allergic diseases in children. Lactoferrin’s multifaceted properties make it a compelling candidate for managing these conditions. Firstly, lactoferrin exhibits potent anti-inflammatory and antioxidant activities, which can mitigate the chronic inflammation characteristic of allergic diseases. Secondly, its iron-binding capabilities may help regulate the iron balance in allergic children, potentially influencing the severity of their symptoms. Lactoferrin also demonstrates antimicrobial properties, making it beneficial in preventing secondary infections often associated with respiratory allergies. Furthermore, its ability to modulate the immune response and regulate inflammatory pathways suggests its potential as an immune-balancing agent. This review of the current literature emphasises the need for further research to elucidate the precise roles of lactoferrin in allergic diseases. Harnessing the immunomodulatory potential of lactoferrin could provide a novel add-on approach to managing allergic diseases in children, offering hope for improved outcomes and an enhanced quality of life for paediatric patients and their families. As lactoferrin continues to capture the attention of researchers, its properties and diverse applications make it an intriguing subject of study with a rich history and a promising future.

## 1. Introduction

The history of lactoferrin (LF) spans several significant milestones. Initially observed in 1939 by Sorensen and Sorensen as an iron-containing red protein in bovine milk, comprehensive characterisation was delayed due to extraction challenges [1]. By 1960, in-depth investigations had elucidated LF’s molecular weight, isoelectric point, optical absorption spectra, and the presence of two iron atoms per protein lobe. Officially named “lactoferrin” in 1961, it was found to share structural and chemical similarities with serum transferrin. Subsequent research expanded understanding beyond its iron-binding properties, revealing roles in iron absorption, metabolism, antimicrobial activities, and infant infection protection [2,3].

**Figure 1:**
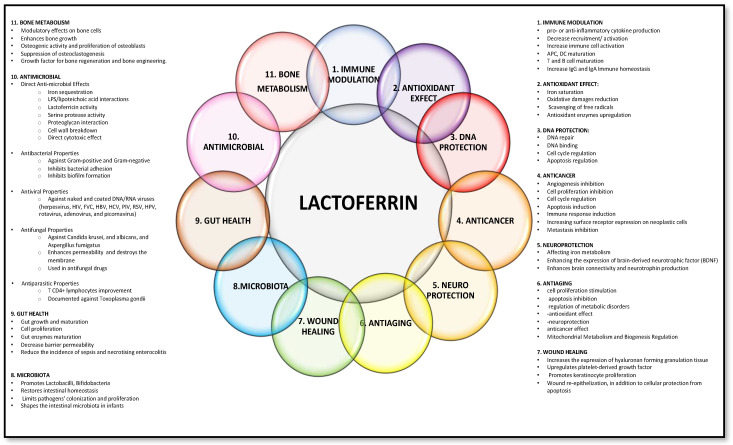
Some of the lactoferrin properties behind the iron-binding ones. Adapted from [6,29,30,31,32].

During the 1970s and 1980s, LF’s therapeutic potential in treating iron-related disorders and infections gained recognition, leading to clinical studies in neonatology and wound healing. The 1990s saw discoveries of its immune-modulating properties, establishing LF as a crucial component of the innate immune system. From the 2000s onward, LF has been extensively applied in the food and pharmaceutical industries [4]. LF has gained significant interest in the health benefits of dietary supplements. Biotechnological advancements have enabled the production of recombinant LF, expanding its research and commercial availability. LF can be used as a principal bioactive agent or to enhance the efficacy of therapeutic drugs, protect drugs from enzymatic degradation, and improve bioavailability in targeted drug delivery systems [5]. Research in the 21st century focuses on LF’s mechanisms, personalised medicine applications, and emerging health challenges, earning it the moniker “miracle molecule” [6]. LF’s potential includes countering pathogens, combating cancer, reducing inflammation, modulating the immune system, and regulating DNA functions. Emerging research highlights its role in addressing neurodegenerative conditions and stress-related disorders [7,8,9]. Additionally, LF has shown innovative roles in protecting and repairing genetic material and modulating the cell cycle [10]. Last but not least, during the COVID-19 pandemic, LF’s antiviral activity against SARS-CoV-2 in vitro and in vivo has been noted, though further studies are needed to confirm its effectiveness [11,12,13,14]. The main LF’s roles in health and diseases are summarised in Figure 1.

LF’s notable role in modulating immune responses in various immune-mediated diseases deserves a separate mention, addressing both endogenous and exogenous “danger signal”(DAM and PAMP)-induced immune responses and adaptive ones [15,16]. Indeed, LF interacts with cell surface receptors crucial for recognising “danger signals” (Toll-like receptor, CD14, and CD22), thereby altering intracellular signalling pathways. This interaction produces a modified subset of proteins that help control and contain the inflammatory response [17,18,19]. For example, in rheumatoid arthritis, LF can inhibit pro-inflammatory cytokines like TNF-α and IL-1β, reducing joint inflammation and damage [20,21,22]. It promotes regulatory T cells (Tregs) in multiple sclerosis, aiding immune tolerance and preventing neurodegeneration [15,23,24]. LF’s ability to modulate gut flora and reduce intestinal inflammation benefits inflammatory bowel diseases such as Crohn’s disease and ulcerative colitis [25,26,27]. Its anti-inflammatory effects modulate autoimmune responses [15]. The multifaceted immunomodulatory properties of LF underscore its capacity to act on a broad range of immune-mediated diseases. It offers therapeutic potential through its ability to balance and adapt the immune response to internal and external threats. Further research is warranted to elucidate these benefits and translate them into clinical applications fully. 

Despite what is mentioned above, the scientific literature shows the lack of studies and clinical trials highlighting its potential in airway allergic diseases or its role in allergen-associated immune responses (AAMPs) [28]. Precisely for this reason, our review aims to investigate LF’s potential as a therapeutic agent in this field, exploring the underlying roles in airway allergic diseases, such as allergic rhinitis and asthma, including its impact on bridging innate and adaptive immunity and interaction with antigens, including allergens. This analysis leads us to assess the findings in the context of the existing literature, speculate, and draw conclusions about this interesting glycoprotein.

## 2. Genetics and Molecular Structure

Understanding the genetic features of LF is essential for researchers to investigate its roles and potential genetic variations that may impact LF’s functions, including in the allergy field. LF is encoded by the lactoferrin (LTF) gene on human chromosome 3 (3p21.31). The sequence shows a high level of conservation across human, mouse, bovine, and porcine species. Out of 17 exons, 15 encode the same number of amino acids in these species, and in 12 specific locations, they have similar codon interruptions at the intron–exon splice junctions. However, LF expression is ubiquitous and species-, tissue-, and cell-type specific. Produced by exocrine glands, this substance is found in mammalian milk, saliva, and tears, in secretions from the intestines and airways, and the secondary granules of neutrophils. It is differentially regulated through multiple signalling pathways such as steroid hormone, growth factor, and kinase cascade [33]. 

**Figure 2:**
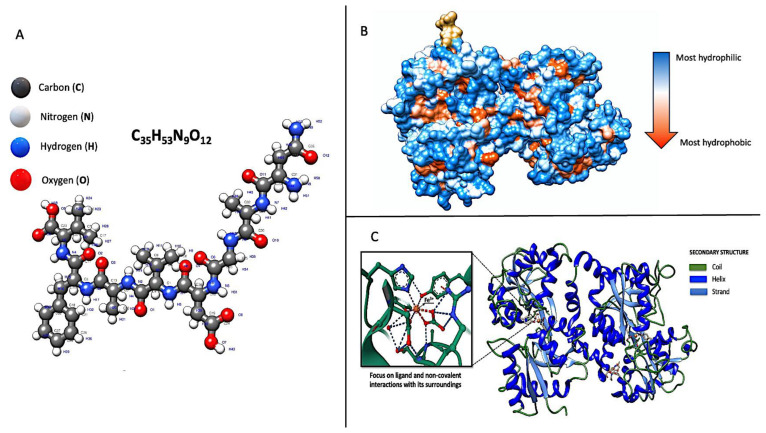
Molecular features of human lactoferrin. (**A**) Chemical structure. (**B**) Hydrophobicity surface: from dodger blue for the most hydrophilic, to white, to orange-red for the most hydrophobic. (**C**) Iron-saturated secondary structure and focus on iron-binding site. From [34,35].

Indeed, LF is characterised by several genetically determined molecular features contributing to its structure and function, as summarised below (Figure 2):

Protein Structure: LF comprises a cationic single polypeptide chain with a molecular weight of approximately 80 kDa. It consists of two symmetrical lobes, the N-lobe and the C-lobe, which are connected by a hinge region. Each lobe, chelating with high affinity to a single Fe^3+^ ion, contains two domains: the N-terminal and the C-terminal domain [36]. Various environmental conditions, including temperature, pH levels, ionic strength, and other proteins and polysaccharides, influence the molecular stability of LF and its tendency to denature [37], determining its nutritional and therapeutic value. For example, LF is capable of interacting with negatively charged proteins. In milk, roughly 50% of the LF forms complexes with negatively charged casein micelles via electrostatic forces: upon introducing LF to milk, its binding to casein micelle surfaces initially leads to an increase in the micelles’ size and opacity, subsequently followed by the breakdown of the casein micelles [38]. Heat treatment facilitates the creation of complexes between LF and casein when preparing infant milk formula by attaching the LF to casein. This connection significantly alters the thermal stability of the LF, leading to its faster thermal denaturation compared to the LF in a pure protein solution [39,40]. Goulding et al. showed that heating alters its original secondary LF structure, specifically, a decrease in α-helix regions and an increase in intermolecular β-sheet formations. This thermal processing also causes lactoferrin to lose colour, increases its surface hydrophobicity and cationic surface charge, and creates protein–protein aggregates linked by disulfide bonds [41]. While LF possesses numerous biological functions, maintaining these functions in processed foods is challenging. Studies have shown that forming complexes with pectin or soluble soybean polysaccharide can shield LF from heat-induced damage and preserve its ability to bind iron [42,43]. Moreover, polyphenol compounds that can bind with LF were found to inhibit the thermal aggregation of LF by increasing steric hindrance and electrostatic repulsion [44]. Sodium caseinate also emerges as a promising option to enhance LF’s heat stability through the development of stable complexes and by binding to LF’s exposed hydrophobic groups, thus preventing the thermal aggregation of LF molecules [38]. Studies have also indicated that the disparity in denaturation temperatures between holo-LF (iron-saturated LF) and apo-LF (iron-free LF) stems from the tighter, more compact structure of holo-LF resulting from iron binding. Thus, iron saturation is crucial in enhancing its durability against heat-induced denaturation [45,46,47]. 

Glycosylation: Glycans are one of the four fundamental classes of macromolecules inherent to living systems, alongside nucleic acids, proteins, and lipids. These structures comprise monosaccharide units linked by glycosidic bonds in diverse ways. Enveloping every living cell, glycans are essential to all forms of molecular communication, enabling cellular sociability. They play multiple roles in molecular recognition, immunity, and inflammation, as they are covalently attached at specific sites on many eukaryotic proteins, thus modulating aspects of their biological activity or influencing their circulation lifespan in the bloodstream. Typically, proteins are glycosylated at various points via an N-glycosidic bond by an oligosaccharide, beginning with glucosamine. These oligosaccharides, known as N-glycans, possess a common backbone due to their shared biosynthetic pathways [48]. Glycans can affect cellular reactions in two primary ways: directly, through receptors that specifically recognise a glycan, or indirectly, by altering the conformation of the molecule to which they are attached. LF is glycosylated. It is adorned with bioactive oligosaccharides linked to specific amino acid residues that, depending on its varied glycosylation profiles, serve as post-translational modifications that influence LF’s functionality [49]. Thus, the amino acid sequence can predict possible N-glycosylation sites. The glycosylation patterns can vary depending on the species, as well as on the source (cell type) and the physiological status, and, in turn, influence LF’s biological activities, stability, and interactions with other molecules [49]. There are three potential glycosylation sites in human LF, while in bovine, there are five [50]. Considering these features, it is easy to understand how the origin of endogenous LF can influence its activity and how supplementation with exogenous (e.g., bovine or recombinant LF) may modify its bioactivity. Intriguing research, based on the assumption that children raised on farm exhibit a reduced risk of developing atopic diseases, examined the quantitative glycoproteomic of human milk samples from Old Order Mennonite (OOM) mothers with rural lifestyles and the risk of atopic disease compared with the milk samples from Rochester urban/suburban mothers. Among the 24 N-glycopeptides of LF identified, the abundances of nine glycopeptides were significantly different between the samples from the mothers in Rochester and OOM. The results of this study indicate that variations in the glycosylation of milk proteins may be associated with atopic disease.

Iron-Binding Sites: Despite the different origins of LF, the iron-binding sites are all roughly the same. It contains two high-affinity iron-binding sites, one in each lobe, that reversibly bind one iron ion each. Only the C-lobe is bound to iron ions when iron ions are not saturated, indicating that LF releases iron ions from the N-lobe first. These iron-binding sites are crucial in LF’s functions, including its antimicrobial, antioxidant, and immunomodulatory activities [37]. The chelation process, which mitigates iron overload, is vital because excess iron can be detrimental. It may transfer electrons to oxygen, creating reactive oxygen species (ROS)-like superoxide anions and hydroxyl radicals [51]. Indeed, LF is known to counteract the process often called the oxygen burst in neutrophils, generating substantial quantities of free radicals and harming cells and tissues [52]. Additionally, its iron-binding capacity reduces its availability to pathogens dependent on iron for their growth [6]. For example, LF binds one ferric iron atom in each of its two lobes and does not release it even at pH 3.5. This is important as that property ensures iron sequestration in infected tissues where the pH is commonly acidic [53]. In recent years, researchers have observed the low pH values of Airway Surface Liquid in asthma and allergic rhinitis, implying that a correct conformation and function of LF could contribute to protection from infectious complications through the mechanism just described. However, it is a common experience and scientific evidence that there is an increased susceptibility to infections in atopic pathologies due to altering multiple and intricate mechanisms [54]. A compromised epithelial barrier not only facilitates the transmucosal entry of pathogens but also sensitisation to aeroallergens can trigger polarised Th2 inflammation, disrupt IgE regulation, remodel airways, and alter neural responses, thereby intensifying local inflammation and weakening both the epithelial barrier and its defence against microbes. In allergic inflammation, notably in allergic children, there is an increased expression of ICAM-1, the primary receptor for rhinovirus, heightening tissue vulnerability to rhinovirus infections. Additionally, the release of IL-13 during allergic airway inflammation impacts ciliary beat frequency, further enabling the viral invasion of the nasal mucosa [54]. In addition, it is natural to wonder if this iron sequestration mechanism may have a long-term influence on iron metabolism in allergic patients’ chronically inflamed respiratory epithelium. 

Antimicrobial Regions: LF has specific structural regions that exhibit antimicrobial properties. These regions in the N-terminal domain are responsible for LF’s ability to inhibit various microorganisms, including bacteria, fungi, and viruses. LF was initially thought to have an indirect role in host defence because of its ability to sequester iron needed for bacterial survival. This bacteriostatic mechanism was supposed to be LF’s primary function until it was demonstrated that iron-free LF had bactericidal activity independent of iron binding [55]. LF can directly engage with microorganisms, offering a range of defensive actions. For bacteria, it enhances phagocytosis, suppresses biofilm formation, and hinders LPS-mediated activation while also altering the interactions between microbes and host cells [32,56]. Specifically, in the case of the fungus, as observed for Candida albicans, LF can trigger apoptosis [57]. Its antiviral properties are demonstrated by mitigating virus-induced cell apoptosis and obstructing the virus’s entry into cells. This is achieved by binding to proteins on the viral envelope or viral receptors on cell surfaces [56,58,59].

Binding Sites for Ligands: LF can bind to various ligands, including iron, lipids, carbohydrates, and microbial components. It contains specific binding sites within its structure that enable these interactions, allowing LF to participate in diverse biological processes [33]. In addition, LF can chelate other metal ions, including Al^3+^, Cu^2+^, Mg^2+^, Mn^3+^, Zn^2+^, and Ca^2+^, through the same binding site, though its affinity for these ions is lower than Fe^3+^ [60]. These interactions highlight its role in regulating metal homeostasis and its potential as a transporter for these metals. A critical yet unresolved question is how bovine LF in infant formula interacts with chemically supplemented ions. There is limited research on whether LF supplemented with additional ions alters its biological activity, particularly in immune cells [61]. For instance, studies on the impact of Cu^2+^-supplemented bovine lactoferrin (bLF) on immune cells, like murine splenocytes and macrophages, revealed that Lf with low Cu levels affected these cells differently than LF alone. This included a reduced suppression of splenocyte proliferation, enhanced macrophage activation, an increased CD4+/CD8+ ratio in T lymphocytes, and the secretion of specific cytokines, like tumour necrosis factor-α (TNF-α), interleukin-6 (IL-6), IL-1β, and inflammatory mediators namely prostaglandin E2 (PGE2), nitric oxide, and ROS [62,63]. More specifically, Cu-fortified bLF products, at the concentrations of 10 and 20 μg/mL, affected stimulated cells differently, altering the release of inflammatory mediators based on the Cu concentration and dosage. Lower Cu levels (0.16 mg/g LF) and doses (10 μg/mL) in LF showed increased anti-inflammatory effects. In contrast, higher Cu levels (0.32 mg/g LF) and doses (20 μg/mL) led to reduced efficacy in lowering inflammation. The activity of LF, either enhanced or diminished, is directly influenced by the Cu level, suggesting that native LF’s potential activity is also modified when combined with intentionally added Cu(II) in dairy products [63]. These observations indicate that Cu^2+^ supplementation can influence LF bioactivity, with the Cu^2+^ level being pivotal in determining immune cell function [62]. Further studies are essential to decode the mechanisms behind these effects. Nonetheless, more research is required to fully comprehend the mechanisms and implications of such interactions, especially regarding human health and allergic diseases. For example, extensive studies have demonstrated that allergic inflammation decreases intracellular zinc levels and changes the gene expression of zinc transporters [64,65]. Moreover, it is believed that zinc release through airway secretions increases during inflammatory conditions, potentially exacerbating zinc deficiency [66]. Since zinc deficiency intensifies inflammatory or immune responses, these observations suggest a possible vicious cycle between zinc deficiency and allergic inflammatory states [67]. Interestingly, human LF can also modulate zinc absorption in the intestine [18,19]. Thus, complexes based on LF should have several beneficial effects on the organism and seem promising in treating zinc and iron deficiency. The relationship between Zinc and LF is more complex. In fact, besides the fact that the mechanisms of zinc binding to hLTF comprise several steps, and the increase in Zn^2+^ concentration intensifies the process [68], an exciting study demonstrated a peculiar effect on the immunomodulatory activity of bFL exerted by Zn supplementation. Commercial bovine LF was fortified with zinc at concentrations of 0.16, 0.32, and 0.64 mg/g LF, achieving zinc saturation of 10%, 20%, and 40%, respectively, while unsaturated bovine LF served as the control. The fortified LF samples were tested in vitro on two types of immune cells: murine splenocytes and RAW264.7 macrophages. The findings indicated that the standard bovine LF and its zinc-supplemented variants suppressed activity in splenocytes and those stimulated with concanavalin A (ConA) and lipopolysaccharide. However, at lower zinc saturation levels and smaller doses, this suppression was mitigated and, in some cases, reversed. Notably, zinc-supplemented LF at lower doses and saturations slightly increased macrophage stimulation, enhanced the CD4+/CD8+ ratio of T lymphocyte subpopulations, and significantly boosted cytokine production (IL-2, IL-4, and INF-γ in splenocytes, and IL-1β, IL-6, and TNF-α in macrophages). In contrast, the higher doses and saturations of zinc in LF tended to produce opposite effects in these cellular models. These results suggest that zinc supplementation significantly influences the immunomodulatory effects of bovine LF, with the level of zinc saturation playing a crucial role in modulating immune responses [69].

Understanding the molecular structure and features of LF is crucial not only for unravelling its mechanisms of action and designing LF-based therapeutics and interventions in atopic diseases but also for understanding how the genetically derived alterations of these functional domains can compromise its functionality, contributing to the pathogenesis of atopic diseases. Like many genes, the LF gene can also exhibit genetic variation among individuals. These genetic variants can influence its levels or functionality. They may affect health and disease susceptibility by impairing LF’s ability to bind iron, its antimicrobial properties, or interactions with the immune response [70]. Some single nucleotide polymorphisms (SNPs) have been identified and have been associated with various conditions: for instance, two polymorphisms in the first exon of the LF gene, resulting in amino acid changes (Thr29Ala and Arg47Lys) in the N-terminal region of the protein that mediates its antibacterial properties have been associated with different pathologies, such as periodontitis [71,72,73], dental caries [74,75], ovarian cancer [76], nasopharyngeal carcinoma [77], dyslipidaemia [17], and coronary artery stenosis [78]. However, to our knowledge, no studies exist on the association between LF gene polymorphism and atopic diseases. The reason for this gap in the scientific literature is not known. Instead, a few reflections are enough to understand how potentially important it could be to investigate the association between the polymorphisms of the LF gene and atopic diseases to understand their pathogenesis and complications better. LF interfaces with the external environment in healthy individuals and plays a significant role in immune and inflammatory responses, immune modulation, and cell growth promotion [55]. Research findings have revealed that normal human tissues exhibit varying levels of LF expression across different organs and under diverse physiological and pathological circumstances. However, LF is reportedly present in modified quantities in diseased tissues compared to its healthy counterpart, indicating a disruption in LF expression [79]. The aberrant expression of the LF gene can be attributed to multiple factors, including mutations in the promoter region or alterations in the gene’s structure. An occurrence of mutation, deletion, or substitution in the regulatory region of the LF gene is anticipated to result in altered expression. Conversely, mutations in the coding regions of the LF gene may lead to an irregular protein structure, consequently influencing its function. Identifying changes in the LF gene within diseased tissues could offer valuable insights into the dysregulation of the LF gene in the context of atopic conditions [80]. 

Previous observations refer to an altered expression of LF in inflammatory diseases, massively released from neutrophil secretory granules in the inflamed tissue [55]. For instance, in allergic rhinitis, the levels of mucosal LF are increased to indicate its possible use as a biomarker: the levels of LF in nasal lavage fluids increased significantly after the nasal provocation test with Dpt (*Dermatophagoides pteronyssinus*) in an independent manner from eosinophil cationic protein levels, inflammatory cells counts, and disease severity evaluated with total nasal symptom scores. In addition, the combination of serum lactoferrin level and serum Dpt-specific IgE level was helpful as a serologic marker for the early detection of Dpt-sensitive AR with a sensitivity of about 81.4% and specificity of 79.2% [81]. In addition, despite the evidence of its involvement in allergic asthma, it is unknown whether neutrophils can be one of the primary cellular sources of this critical inflammatory mediator directly in response to an IgE-mediated stimulus [82,83]. Experimental research shows the ability of human neutrophils to release LF in response to allergens, which induce positive skin prick tests and specific IgE in asthmatic patients. The amount of its release was correlated with serum-specific IgE levels and the severity of allergic asthma symptoms, pointing out a possible use of this protein as a follow-up tool to measure the progression of the allergic condition and the effectiveness of treatment [83]. The concentrations of circulating LF in atopic dermatitis were also significantly higher than in healthy donors, suggesting a remarkable role in the pathogenesis of other atopic diseases [84]. Still, whether this altered expression results from inflammation as an adaptive mechanism or a genetically determined qualitative and quantitative altered expression is unknown; what is certain is that exogenous administration is necessary to improve the clinical status of atopic patients [85]. Still, the alteration of endogenous LF could contribute, as already demonstrated in chronic rhinosinusitis with nasal polyposis (CRSwNP). The best-described deficiency in innate immunity associated with nasal polyposis is a deficiency in mucosal LF production [86]. A Polish research group observed a significant association between the 140 A/G polymorphism of LTF and an increased risk of developing CRSwNP, which is more substantial in patients with allergies or asthma than those without these conditions [87]. In addition, we must consider that LF can provide the environmental stimulus to stabilise the maintenance and restoration of the resident microflora. Over the last several years, it has become more apparent that atopic diseases involve a community of bacteria that move from health to disease by a shift in the qualitative and quantitative composition of members of the resident flora, such as a dysbiosis characterised by impaired biodiversity. The shifts can occur due to ecological stimuli caused by numerous physical, chemical, and biological influences [88]. The genetic variants of LTF may contribute to developing the dysbiosis typical of atopy as a critical determinant of microbial community composition and behaviour.

Given the complexities depicted above and the limited database assessed, it is mandatory to investigate specific genetic assets that show biological plausibility relative to disease pathogenesis and genome-wide association studies. This strategy may allow the emergence of new diagnostic and therapeutic approaches to atopic diseases in the future [55]. All this without even considering LF’s role in immune responsiveness and immunomodulation, as shown below. 

## 3. Lactoferrin Receptors

Lactoferrin plays its role by interacting with specific receptors (LfRs) in various tissues and cell types, including hepatocytes, kidney cells, lymphocytes, monocytes, and different brain areas [89]. Here are only some valuable examples.

**Figure 3:**
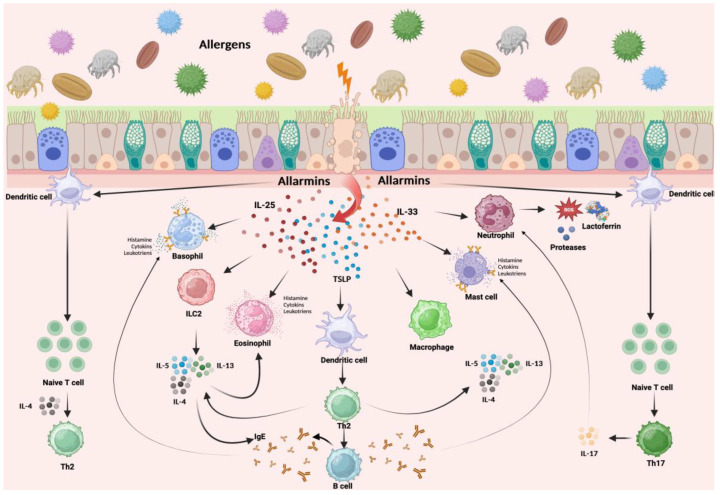
Illustration of the common airway allergic inflammatory cascade that depicts a sensitised individual’s complex immune response to allergens. Allergens trigger allergic reactions from the damaged epithelial layer barrier that releases alarmins, such as TSLP, IL-25, and IL-33. Essential immune cells involved in the allergic response include dendritic cells, basophils, ILC2s (type 2 innate lymphoid cells), eosinophils, mast cells, macrophages, T cells (Th2 and Th17), and B cells producing IgE antibodies specific to the allergens. The interactions between these cells are mediated by cytokines such as IL-4, IL-5, IL-13, and IL-17, which promote inflammation and the allergic response mediated by histamine, cytokines, ROS (Reactive Oxygen Species), and other mediators. The network of interactions ultimately leads to allergy symptoms and tissue remodelling. Created with https://BioRender.com (URL accessed on 11 April 2024).

Intelectin 1 (ITLN1) is a type of lectin that plays a vital role in the body’s innate immune response and acts as a high-affinity receptor for LF [90]. While it is primarily located in the intestinal brush border, ITLN1 is also found in other cell types such as Paneth and goblet cells [91], the biliary epithelium [92], as well as being notably present in the airway epithelium and in the skin keratinocytes [93]. In the complex environment of the respiratory tract, ITLN1 is primarily involved in recognising and binding to pathogen-associated molecular patterns (PAMPs). This interaction is crucial for initiating the body’s immune response to pathogens, binding with carbohydrates expressed on the surface of bacteria, viruses, and fungi as players in the innate immune response. However, the mechanisms of its production and function are still controversial. Indeed, the presence of ITLN1 in the airways is particularly significant given the high respiratory tract exposure to environmental particles and pathogens. Still, its role extends beyond mere pathogen recognition. More interestingly, research has indicated that a specific single-nucleotide polymorphism in ITLN1 is linked to an elevated risk of asthma [94]. Additionally, the expression of ITLN1 is heightened in mice with ovalbumin (OVA)-induced allergies and in mice with an overexpression of IL-13 [95,96]. ITLN1 plays a significant role in promoting eosinophilic inflammation in the airways of OVA-allergic mice. Indeed, a previous study reported that airway eosinophilic inflammation and mucus production were inhibited after airway transfection with ITLN shRNA (short hairpin RNA), an RNA sequence that folds back on itself to form a structure resembling a hairpin, used to silence genetic expression through the activation of RNA interference, in an OVA asthma model [97]. Given ITLN’s role in the innate immune response, it is theorised that ITLN experiences an upsurge in the early stages following allergen sensitisation in the airways. This upregulation is believed to be crucial for the expression of IL-25, IL-33, and TSLP [93]. These are critical epithelial and epidermal innate cytokines instrumental in triggering the onset of allergic inflammation cascade observed in conditions like allergic rhinitis and asthma (Figure 3) [98,99]. A fascinating study revealed that in mice with significantly reduced ITLN expression, or ITLN knockdown (KD) mice, symptoms such as airway hyperresponsiveness (AHR), eosinophilic inflammation, mucus overproduction, and type 2 immune responses were significantly diminished, not only in the OVA-induced asthma model but also in a house dust mite (HDM)-induced asthma model [93]. Further examination of ITLN‘s involvement in the early expression of IL-25, IL-33, and TSLP before evident airway inflammation occurred involved mice intranasally exposed to HDM for three consecutive days. Il-25, Il-33, and TSLP expressions were initiated 2–6 h after the final HDM exposure, with the ITLN1 expression being triggered early (at 2 h) after the last HDM exposure. Nonetheless, in the ITLN KD mice, the HDM-induced expressions of IL-25, IL-33, and TSLP were inhibited. In vitro experiments showed that HDM stimulated the expression of ITLN1, IL-25, IL-33, and TSLP in human bronchial epithelial cells within 2–6 h. However, this expression was reduced when ITLN1 was suppressed through RNA interference or when the ITLN1 activity was blocked by adding galactose [93]. These results suggest ITLN’s potential role in the innate immune response to allergen exposure in the airways, facilitating the expression of IL-25, IL-33, and TSLP, aligning with studies showing increased ITLN1 levels associated with the sputum eosinophilia of asthma patients during flare-ups [96]. Besides initiating type 2 immune responses, ITLN functions as an effector molecule, with its expression being a result of these type 2 responses. Its expression rapidly escalates in mouse lung tissue and human bronchial epithelial cells following stimulation with IL-13, a cytokine associated with type 2 responses [100]. Given the twofold involvement of ITLN, both initiating and responding to type 2 immune processes, it likely plays a significant role in intensifying these responses in diseases like allergic ones. Moreover, while it is true that reducing ITLN1 levels has been observed to decrease the expression of Th2 cytokines in response to allergens, prior studies indicate that artificially elevating ITLN levels in type 2 pneumocytes does not trigger type 2 immune responses [101]. This implies that the effectiveness of ITLN might depend on additional factors activated during the innate immune response. The role of lactoferrin in this context is not known to date. Still, it would be interesting to verify whether this protein, characterised by high affinity to ITLN1, can inhibit the allergen-related activation of ITLN1 and the existence of ITLN1 polymorphism related to allergic diseases. 

The monocyte differentiation antigen 14 (CD14), a 55 kDa glycoprotein, exists as a soluble serum protein (sCD14) and a GPI-anchored protein (mCD14) on monocytes–macrophages [89]. Among the genes linked to asthma, the CD14 gene is one of the most extensively researched. This gene is responsible for coding a protein that acts as a co-receptor for the Toll-like receptor (TLR) and plays a crucial role in the innate immune response. The CD14 receptor, known for its multifunctionality and high specificity for LPS, forms a complex with TLR4. This complex triggers the innate immune system through various pathways, releasing pro-inflammatory cytokines such as interleukin-1 (IL-1) and tumour necrosis factor-alpha (TNF-α). The CD14 gene is on chromosome 5q31.3, a region associated with asthma in genome-wide linkage studies [102]. LF’s interaction with the CD14 receptor effectively competes with bacterial LPS. It can decrease the transcription of genes for various inflammatory mediators induced by NF-κB, acting as a regulatory agent in the mechanism of acute inflammation [15]. Human lactoferrin (hLf) can attach to sCD14 and sCD14-LPS complex, suppressing specific cellular activation processes, precisely ICAM-1 and E-selectin expression. These molecules are critical for the recruitment of leukocytes. Additionally, hLf’s interaction with CD14 could explain its documented influence on the production of proinflammatory cytokines such as tumour necrosis factor-alpha, IL-1, and IL-6. Consequently, hLf might regulate the attraction of immune cells to the sites of inflammation and may be released by neutrophils in response to inflammatory conditions [103]. However, the role of CD14 in atopic disease is not clear. Experimental studies indicate that lowering CD14 levels can decrease the expression of Th1 cytokines (like IL-12 and IL-18) triggered by pathogens, thereby favouring Th2 differentiation within adaptive immunity [104,105]. Conversely, antigen-presenting cells activated by bacterial ligands exhibited increased CD14 expression. When combined with the presentation of allergens by these immune cells, this enhanced expression can lead to reduced Th2 differentiation and the suppression of IgE responses [104]. These findings suggest a connection between single nucleotide polymorphisms (SNPs) in the CD14 gene and atopy [102]. A specific genetic marker within these, the Cytosine-to-Thymine substitution at position -159 in the CD14 promoter, has been linked to serum sCD14 and total IgE levels [89]. This association between CD14/-159, serum total IgE levels, and atopy was observed only in skin-test-positive children and adults [104,106,107]. However, previous studies by Gao and colleagues identified a different association in British subjects, with CD14 -159C linked to higher serum total IgE levels in those negative for allergen-specific IgE [108]. Further complicating matters, a genome screen of the Hutterites linked the T allele at CD14/-159 to atopy [109], while a subsequent study found no association [110]. These varying results could be due to gene–environment interactions, where CD14 polymorphisms, altering the interaction with LPS and favouring Th2 differentiation, may act as a modifier favouring atopy in individuals exposed to different levels of LPS, however difficult to quantify. Adding to this complexity, a complementary study showed that the reduced levels of soluble CD14 in maternal breast milk and its exposure to the fetal and neonatal gastrointestinal tract are linked to developing atopic diseases [111,112]. Thus, an exogenous intake of sCD14 might affect immunologic homeostasis locally and systematically in early life, influencing allergic disease outcomes. Amidst these complexities, the delicate balance between inflammation and genetic and environmental elements highlights the necessity for thorough research into how gene variations related to innate immunity contribute to atopy-related disease pathology. This also underscores the importance of understanding the role of lactoferrin in these complex pathways, particularly in allergic diseases and the combinative role of CD14 and lactoferrin in breast milk.

To summarise, LF exhibits an affinity for receptors associated with “danger signals”. It alters the type of intracellular signalling through identified receptors like ITLN1, CD14, and others, such as Toll-like receptors (TLR) 2 and TLR4. This produces a subset of proteins that help manage and limit the inflammatory response [15]. Additionally, LF may influence other signalling receptors, such as RAGE or TREM-1, which activate NF-κB pathways, or Nucleolin, a 105 kDa nucleolar protein pivotal in transcription regulation, cell proliferation, and growth, which is a transporter between the cell surface and the nucleus, suggesting hLF involvement in nuclear process regulation [15,61].

The range of biological functions exhibited by LF is contingent on the types of cells it targets and the availability of specific receptors on their surfaces. Numerous receptors indicate the broad and diverse impacts that LF can potentially exert. However, more research is needed to fully understand these mechanisms in allergic diseases and translate these findings into clinical applications.

## 4. Lactoferrin as “Middleman” of Innate and Adaptive Immunity

LF influences the immune system through multiple interactions, impacting the functioning of various immune cells, including macrophages, dendritic cells, B cells, and T lymphocytes, and affecting the production of various cytokines [82]. LF is an effective “middleman” of innate and adaptive responses with a two-pronged behaviour. This mediation consists of local and systemic arrangements in the expression of the signalling molecules that rule the balance between pro- and anti-inflammatory, as well as humoral and cellular immune feedback. In general, several investigations underscore that LF plays a significant role in modulating cytokine activity via the upregulation of anti-inflammatory cytokines like IL-4 and IL-10 and the regulation of pro-inflammatory cytokines such as NO, TNF-α, IL-1, IL-6, IL-8, and granulocyte–macrophage colony-stimulating factor [16,23,113,114,115]. In addition, LF has been shown to interact with the complement system by inhibiting its activation, thereby reducing the inflammatory response associated with complement activation [116]. The complement system plays a crucial role in the immune response, particularly in inflammatory diseases like asthma, where complement activation can exacerbate inflammation and tissue damage. Studies have demonstrated that complement system components, such as complement factor H (CFH), play critical roles in modulating immune responses and inflammation in asthma [117,118]. CFH is a key regulatory protein in the complement system, specifically regulating the alternative pathway. It prevents overactivation and subsequent tissue damage by binding to C3b and facilitating its inactivation. This action halts the complement cascade, preventing the formation of the C3/C5 convertase, which would otherwise lead to opsonisation, inflammation, and cell lysis [118,119]. LF’s interaction with CFH is fascinating in the context of immune regulation. Both proteins modulate the complement system, albeit through different mechanisms. While CFH prevents unnecessary inflammation, lactoferrin binds to complement system components, such as C3b and C4b, inhibiting their activity. This inhibition can reduce the formation of the membrane attack complex (MAC) and subsequent cell lysis [120]. In diseases like asthma, where inflammation and immune dysregulation are prominent, the combined and synergistic effects of CFH and LF could be particularly beneficial. CFH can mitigate the activation of the complement system, reducing inflammation and tissue damage. Concurrently, LF can further suppress inflammatory responses and promote immune homeostasis. This dual modulation can improve clinical outcomes by simultaneously addressing the multiple pathways involved in the allergic disease process. Furthermore, studies have shown that LF promotes CD4+ T cell differentiation, leading to an increase in the Th1/Th2 cytokine ratio, thereby enhancing the type 1 immune response and elevating the expression of type 1 cytokines, such as IFN-γ and IL-12, with in turn, downregulating allergic reactions typically driven by a type 2 response [121]. While many others potentially remain the subject of ongoing research, a limited number of molecular mechanisms have been identified. More specifically, as we mentioned above, many immunomodulatory effects of lactoferrin stem from its capacity to bind various pro-inflammatory PAMPs on innate immune cells. These interactions have been demonstrated to suppress LPS-triggered cell activation at the sites of inflammation, leading to a decreased production of potent pro-inflammatory cytokines. Similarly, endothelial cells’ production of adhesion molecules (E-selectin and intercellular adhesion molecule-1) and IL-8, a crucial element of the chemokine gradient in inflamed tissues, is affected. Consequently, lactoferrin released by polymorphonuclear neutrophils (PMNs) can alleviate responses and subsequently attract immune cells to the core of inflammatory sites, thereby minimising additional tissue damage [114]. In this context, the capacity of LF to blockade eosinophil migration contributes to the suppression of allergic inflammation [122]. However, LF also shows duplicity in its behaviour concerning its chemotactic functions. Indeed, LF serves as an alarmin, facilitating the recruitment and stimulation of antigen-presenting cells (APCs) and eliciting antigen-specific immune reactions [123,124], triggering T-cell activation by influencing dendritic cell functionality, thereby establishing a connection between innate and adaptive immune systems [58,114,123]. The migration of dendritic cells in response to antigen stimulation plays a pivotal role in facilitating antigen-specific immune responses. Alongside its effects on macrophages, including the reduction in pro-inflammatory cytokines and the induction of type I interferon (IFN α/β), as well as impacting their capacity to present antigens to antigen-specific CD4+ T-cells within the adaptive immune system [89,125,126], LF demonstrates its influence on cells that play a key role in identifying antigens and steering the development of adaptive immunity. Also, the interaction between LF and its receptors on T- and B lymphocytes is notably significant. The structural alterations in the N-terminal region of LF and the molecule’s basic properties enhance its interaction with B lymphocytes [127,128]. The oral administration of LF is known to boost the secretion of IgA and IgG in the mucosa of the mouse intestine, indicating LF’s action on B-cells, recognised for presenting antigens, thereby facilitating their engagement with T cells and supporting an increase in non-IgE antibody responses [129,130]. The influence of LF on various T-cell subsets is distinct, with LF able to modulate the allergic inflammatory pathway acting on Th2, Th17, and regulatory T cells (Tregs), mitigating inflammatory reactions. LF fosters Th1 responses while suppressing Th2 responses and induces T-cell receptor cross-linking, leading to diminished T-cell activation and a lower release of allergenic inflammatory mediators like IL-5 and IL-17, thereby lessening inflammation [131,132]. These outcomes have been noted in in vivo experiments utilising dietary lactoferrin, whether derived from the same species (homologous) or a different one (heterologous). While the exact mechanisms remain unclear, it is speculated that these positive effects might stem from lactoferrin’s direct interactions with gut-associated lymphoid tissue, potentially through the receptor-mediated pathways or indirectly through its impact on the balance of gut microbiota [114]. Instances of intact lactoferrin undergoing transcytosis have been documented in preterm infants, neonates, and even adult mice [133,134]. Regardless of the underlying processes, dietary lactoferrin can shape systemic immune responses by altering cytokine levels in the blood and lymphatic system [114]. Significantly, the dietary lactoferrin adjustment of crucial cytokines can influence the Th1/Th2 equilibrium, directing T-helper cell responses towards either anti-inflammatory or pro-inflammatory outcomes.

## 5. Lactoferrin’s Protective Effects against Oxidative Damage

Oxidative stress, characterised by an overload of reactive oxygen and nitrogen species, is critical in developing allergic disorders like rhinitis, asthma, and atopic dermatitis [135,136,137]. This stress damages proteins, lipids, and DNA and can be caused by inflammation and environmental factors like air pollution and cigarette smoke. Antioxidant enzymes in the lungs and their interaction with nitric oxide suggest a complex role in protecting against oxidative damage and cell signalling. The interplay between inflammation and ROS can perpetuate tissue injury through a positive feedback loop, with numerous cytokines activating oxidases that increase ROS levels [138]. LF mitigates excessive inflammatory responses by reducing oxidative stress at the molecular level, acting as an antioxidant due to its capacity to bind iron [139,140]. This iron-binding mechanism allows LF to maintain the physiological balance of ROS and shield cells from oxidative harm [30]. LF triggers the activation of antioxidant enzymes such as catalase, glutathione peroxidase, and superoxide dismutase (SOD), effectively lowering ROS levels [141]. These enzymes collaboratively neutralise radicals, with SOD converting the superoxide radical into hydrogen peroxide, which is then transformed into water and oxygen by catalase and glutathione peroxidase. However, a non-enzymatic reaction occurs with excessive free ferric ions (Fe^3+^). Initially, the superoxide radical reacts with Fe^3+^, producing ferrous ions (Fe^2+^) and oxygen. Subsequently, Fe^2+^ interacts with hydrogen peroxide, producing ferric ions (Fe^3+^), alcohol, and hydroxyl radicals; this is the so-called Fenton reaction [15]. These hydroxyl radicals are particularly hazardous as they can cause lipid peroxidation by reacting with polyunsaturated fatty acids, generating toxic hydroxyalkenals and compromising the integrity of critical biomolecules. Lactoferrin intervenes early in this process by binding to Fe^3+^ ions, preventing the initiation of the reaction and subsequent biomolecular damage. Moreover, LF directly safeguards DNA by neutralising hydroxyl radicals, possibly through a unique interaction with oligonucleotides, thus offering protection against direct oxidative damage [15,139]. While targeting oxidative stress may not resolve allergic diseases for multifactorial reasons, the antioxidant properties of lf could serve as a valuable supplementary therapy in managing them.

## 6. Lactoferrin Clinical Application in Allergic Airway Diseases

Due to its heterogeneous properties, LF has demonstrated several, sometimes conflicting influences in vitro and in vivo experiments in the complex framework of atopic diseases, encompassing several clinical applications involving LF in principal allergic diseases. The studies considered for inclusion were those published in English from 2004 to 2024. A subcommittee determined keywords to formulate the search strategy for each research question.

### 6.1. Allergic Rhinitis

#### 6.1.1. Materials and Methods

A comprehensive electronic search was performed in Pubmed and Cochrane Library databases (Figure S1). Keywords to formulate the query string for the research question were determined by a subcommittee. The research terms used for allergic rhinitis were: ((“lactoferrin”[MeSH Terms] OR “lactoferrin”[All Fields] OR “lactoferrins”[All Fields] OR “lactoferrin s”[All Fields] OR (“lactoferrin”[MeSH Terms] OR “lactoferrin”[All Fields] OR “lactotransferrin”[All Fields] OR “lactotransferrins”[All Fields])) AND “allergic rhinitis”[All Fields]) AND (2004:2024[pdat]).

#### 6.1.2. Results

Whether LF supplementation can lower the risk of rhinitis, shorten the duration of the condition, or reduce the likelihood of complications remains an open question; there were not enough data (Table 1). A promising study on a murine model of allergic rhinitis sensitised to ovalbumin (OVA) highlighted LF’s preventive and therapeutic potential. The administration of 100 μg recombinant human LF (hLF) intranasally was provided in a mice group 4 h before the OVA-allergen challenge and in another group 6 h after the OVA-allergen challenge in comparison with a control group, untreated, and with an OVA-induced AR group. The experiment showed the anti-inflammatory effects of rhLF, including the lower levels of eosinophils and goblet cells at the nasal mucosa histology and IL-5, IL-17, GATA-3, and ROR-C in the nasal fluid lavage of the treated mice. In addition, rhLF had a boosting effect on endogenous LF production, diminished in the AR mice compared to the other groups at nasal lavage fluids. More interestingly, rhLF administration pre-challenge resulted in a more effective anti-inflammatory response than post-challenge administration, curbing allergic inflammation in the mice. This was achieved by tilting the T cell balance towards a Th1 phenotype out of the allergic Th2 and Th17 ones [131]. Studies on humans showed the efficacy of a multicomponent device containing LF in children, improving AR symptoms evaluated with a Visual Analogue scale and objectivated through Active Anterior Rhinomanometry and Mucociliary Transport Time [142,143]. The clinical application of LF may also be helpful in the diagnosis of AR; as mentioned previously, it has been proposed that LF might serve as an early detection biomarker, given that the combination of serum LF concentration and antigen-specific IgE levels can predict the condition with a suitable sensitivity and specificity [81].

### 6.2. Allergic Asthma

#### 6.2.1. Materials and Methods

A comprehensive electronic search was performed in Pubmed and Cochrane Library databases (Figure S2). Keywords to formulate the query string for the research question were determined by a subcommittee. The research terms used for allergic rhinitis were: ((“lactoferrin”[MeSH Terms] OR “lactoferrin”[All Fields] OR “lactoferrins”[All Fields] OR “lactoferrin s”[All Fields] OR (“lactoferrin”[MeSH Terms] OR “lactoferrin”[All Fields] OR “lactotransferrin”[All Fields] OR “lactotransferrins”[All Fields])) AND “asthma”[All Fields]) AND (2004:2024[pdat]).

#### 6.2.2. Results

Although observations on LF applications are more widely discussed in the scientific literature, especially in recent years, data on asthma are also limited (Table 2). The most recent study demonstrated that high-dose LF at 300 mg/kg markedly alleviated airway hyperresponsiveness (AHR) and inflammation in an OVA-induced asthmatic mouse model. LF treatment improved lung function, as shown by reduced Penh values, decreased inflammatory cell counts in bronchoalveolar lavage fluid (BALF), and suppressed pro-inflammatory Th2 cytokines while increasing anti-inflammatory IL-10 levels. Additionally, LF reduced goblet cell hyperplasia, modulated serum OVA-specific IgG1 and IgE levels, enhanced regulatory T cell numbers, and decreased Th2 cytokine production. In vitro LF influenced T-cell differentiation in the spleen, increasing the number of regulatory T cells (Foxp3+) and decreasing Th2-associated IL-4+ T cells, which suggests a shift towards a less allergic, more regulated immune response. It also led to the reduced expression of CD80 and CD86 on dendritic cells, suggesting inhibited DC activation and a shift toward a less allergic immune response [144]. In addition to the effects of LF on the allergic inflammatory cascade, two highly similar studies assessed the impact of milk-derived hLF on inflammation induced by oxidative stress in ragweed allergen-induced allergic asthma, utilising human respiratory epithelial cells in vitro and murine models in vivo. The first study indicates that lactoferrin LF mitigated increased cellular ROS levels in human bronchial epithelial cells induced by ragweed extract (RWE). Significantly, LF markedly reduced the accumulation of eosinophils in the airways and the subepithelium of intranasally challenged, sensitised mice and inhibited the development of mucin-producing cells. Iron-saturated holoLF exhibited no statistically significant impact compared to not saturated LF on RWE-induced cellular ROS levels and inflammation provoked by RWE [145]. The second study reveals that while LF does not affect the enzymatic activity of NAD(P)H oxidase in pollen grains, which generates superoxide anion, it significantly reduces hydrogen peroxide and lipid peroxide levels in the airway lining fluids and lung epithelium following intranasal challenge with RWE in both naive and sensitised mice. Additionally, a single dose of LF effectively reduced the accumulation of inflammatory and mucin-producing cells in the airways and subepithelial areas and decreased airway hyperreactivity induced by RWE. Also, in this case, there was no observed difference with iron-saturated LF, confirming that the mechanisms behind LF’s reduction of allergic immune responses extend beyond simple iron chelation and the prevention of superoxide dismutation [145]. Indeed, another study observed that regardless of its origin, whether from milk or neutrophil-derived, LF inhibits eotaxin-stimulated eosinophil migration without affecting eosinophil viability: the iron-saturation status of LF did not affect its ability to inhibit migration, indicating that its impact on eosinophil chemotaxis is independent of its iron-chelating properties [146]. Controversially, LF can also induce eosinophilia and airway hyperreactivity, as seen in human LF-treated mice and occupational asthma related to bovine LF inhalation [147,148]. However, similar to what was observed in AR, evaluating a specific proteomic signature in the sputum of asthmatic patients as a biomarker for asthma is possible. In particular, this was characterised by a lower level of LF, especially in the exacerbation-prone subjects compared to nonexacerbators [149]. Finally, in an interesting study based on previous observations and considering that lactoferrin receptors have a ubiquitary expression in the body, including the lung, it is suggested that protein fragments with cell-penetrating capabilities, derived from the N-terminal domain of LF, could potentially enhance drug absorption in the lung. To investigate this proposition, the transport of small protein from the apical to the basal side of the immortalised sub-bronchial human epithelium was examined alone and with the corresponding synthetic peptide of the N-terminal domain of hLF. This synthetic peptide, the so-called Human Stimulus Factor, has been shown to enhance the absorption of the small, inhaled corticosteroid molecule across airway epithelial cell models without compromising barrier integrity. Future research should explore the cell-penetrating effects of LF-derived peptides to boost the therapeutics uptake through the respiratory epithelium [150].

### 6.3. Discussion

LF engagement with its multiple ligands initiates a series of overlapping protective reactions, shaping, shifting, and adjusting immune responses and the production of reactive oxygen species, altering cell phenotype, and reducing immune cell recruitment, modulating cytokine and chemokine production; all these actions impact numerous other immune pathways, ultimately leading to the mitigation of pathological alterations in AR et asthma.

Despite LF’s potential, recent studies on its administration in AR and asthma treatment are scarce, with limited animal and human in vitro studies. The recognised potential of LF for medical applications has expanded beyond its initial medical scope to encompass significant commercial interest in nutrition supplementation, cosmetics, and as a source of iron, despite numerous scientific and ethical considerations, including those about safe administration considering the reported allergic airway inflammation LF-induced [147,148]. Notably, LF is available in commercial spray formulations for nasal, oral, and ocular administration. The proposed actions of LF could benefit from a systemic approach, as this protein impacts a variety of metabolic and regulatory pathways, particularly meriting further examination in paediatric applications. Only the two reported studies evaluated the efficacy of LF administration in AR in children, but it is associated with other components [142,143]. Our manual research has found a patent application and a phase II clinical trial for its use in asthma. Still, we have not found published studies or related research outcomes [151,152]. Although Clinicaltrials.gov lists many ongoing studies with both bovine and recombinant LF, surprisingly, none address allergic respiratory diseases, an omission that remains difficult to explain based on current observations.

## 7. Lactoferrin in Human Milk: Concentration Variability, Impact on Infant Health, and Implications for Allergies

In humans, LF is produced by the epithelial cells of the mammary glands and secreted into breast milk during lactation. Its production is influenced by the hormonal changes during pregnancy and lactation, particularly the increase in prolactin levels. LF levels in breast milk can vary depending on various factors, including the stage of lactation, the mother’s health, and environmental factors [4,153]. However, LF is particularly abundant in human milk, comprising 15–20% of the total protein content, while cow milk has a relatively low LF concentration: 1.5 mg/L in colostrum and 0.5 mg/L in mature milk [154,155]. More specifically, its concentration varies significantly across different lactation stages, with the highest content observed in colostrum (5.5 g/L) and a decrease to 1.5–3.0 g/L in mature milk. This variability reflects LF’s biological functions at newborn and infant development stages. Interestingly, prolonged lactation shows LF concentration stabilising between 4.9 and 5.02 g/L, suggesting a physiological response to the diverse environmental demands of the human baby as it gains nutritional independence [156,157,158,159,160,161]. Research further delineates the concentration of LF in milk from the mothers of preterm and term infants, highlighting a higher LF concentration in preterm transitional milk. This suggests that premature neonates may benefit more significantly from LF’s protective effects against infections, supporting the development and functions of their immune system more robustly than term neonates [157,161,162]. The study of LF concentration concerning maternal factors such as body mass index (BMI), serum albumin levels, the mode of delivery, and iron status revealed no significant correlation, indicating that factors beyond maternal nutritional status influence milk LF concentration [157] and considerable variability in LF content between individuals have been noted, indicating that the synthesis of this protein and its transfer from blood to the mammary gland may not be strictly regulated [158]. Indeed, the role of LF in the context of maternal allergies and its impact on infants further complicates our understanding of its functions. In an interesting research, although allergic mothers exhibited higher serum LF levels than non-allergic counterparts, the difference was not statistically significant. However, LF concentrations in the breast milk of the allergic mothers were notably higher, and the infants of non-atopic mothers displayed no allergic symptoms by age two [163]. On the contrary, the incidence rate of allergic conditions among the infants born to atopic mothers stood at 14%. Further examination revealed that the breast milk of atopic mothers with allergic infants contained LF levels exceeding the allergic group’s average. Authors attributed this effect to the early systemic activation of peripheral blood phagocytes, particularly neutrophils, during the acute phase of seasonal allergic rhinoconjunctivitis before or during pregnancy [163]. This phenomenon points to hLF’s potential compensatory effects in inhibiting allergic inflammations, suggesting a dual role in promoting infant health while possibly increasing the risk of atopic conditions in infants with a strong atopic genetic background from their mothers [163]. LF is a critical component of human milk for promoting immunological competence and enhancing offspring survival. Still, it also plays a pivotal role in the development and maturation of the infant’s gut. LF’s involvement in modulating gut permeability and its trophic effect on enterocytes underscores its significance in the gastrointestinal tract’s development. These effects are crucial for preventing infections and necrotising enterocolitis (NEC) in infants, particularly preterms, by enabling more rapid maturation of the intestinal epithelium [164]. Compared to formula, human milk feeding is associated with decreased intestinal permeability. This suggests that LF may contribute to the gastrointestinal tract’s tropism, promoting a faster proliferation of enterocytes in the developing gut. This process leads to a less permeable environment, reducing the risk of pathogens disseminating into the bloodstream through a leaky gut wall [164]. The study by Buccigrossi et al., which explored LF’s effects on enterocytes using bovine LF (bLF) at equimolar to hLF and hLF in vitro using concentrations of 1 and 100 μg/mL, demonstrated that LF has a concentration-related trophic effect on enterocyte proliferation and differentiation, highlighting its key role in intestinal epithelium development, but also that bLF induced an effect similar to hLF on in human intestinal cells [165]. Numerous research efforts have established a connection between heightened intestinal barrier permeability and the development of food allergies. It has been observed that individuals with atopic conditions exhibit an abnormal reaction to food allergens [166,167,168]. The formation of distinct types of immune complexes in response to antigen exposure is crucial, as this mechanism appears to be at the core of the systemic manifestations of food allergies [169]. The progression of food allergy is marked by increased intestinal barrier permeability, which enables allergens to breach this barrier and activate the immune system in the submucosa. This leads to the secretion of cytokines and inflammatory mediators that further deteriorate the epithelial barrier, creating a vicious cycle that exacerbates intestinal permeability [170,171]. Additional research has indicated that individuals suffering from atopic eczema and bronchial asthma also experience increased permeability of their intestinal epithelial cells [172,173]. Furthermore, as the first organ to receive LF when ingested as a natural compound in milk or added as an ingredient in functional food products, the gastrointestinal tract showcases LF’s ability to modulate the gut microbiota composition [174]. This modulation is significant, as altered gut microbiota composition has been associated with an increased risk of atopic diseases [88]. Therefore, hLF’s influence on the microbiota, although not yet well characterised and that should be widely dealt with separately, may contribute to its potential effects in mitigating atopic disease symptoms, aligning with its observed roles in reducing the risk of infections and supporting immune system development. 

This integrated view underscores hLF’s multifaceted role in infant nutrition and development. As commonly occurs with human proteins, utilising the native source is impractical. Although lactoferrin can be purified and obtained from human milk, increasing its production through human milk is not a commercially or medically feasible option. However, while it is a crucial component for infant health, offering protection against infections and supporting gut microbiota, its elevated levels in the context of maternal allergies hint at a complex interplay between genetics, immune response, and environmental factors. The findings call for a nuanced understanding of hLF’s role in human milk. It advocates for further research to unravel its implications for infant health, particularly the intriguing link to maternal allergies and atopic conditions in infants.

## 8. Limitation of Lactoferrin, Lactoferrin Analogues, and Future Perspective

Despite its numerous beneficial properties, LF also has certain limitations that researchers and clinicians should know. LF is susceptible to degradation and enzymatic breakdown in the gastrointestinal tract, which can limit its absorption and bioavailability [82,175]. This can affect its effectiveness when administered orally as a supplement or in food products. LF can vary in composition and purity depending on its source, such as bovine or human milk. The differences in glycosylation patterns, post-translational modifications, and contaminants can influence its activity and effectiveness. LF is a complex protein that can be expensive to produce in large quantities. LF from natural sources, such as bovine or human milk, can be costly and time-consuming. The need for large-scale production methods and purification techniques can pose challenges for commercial applications. Maintaining the stability of LF is crucial to ensure its effectiveness and shelf life to ensure optimal performance for its applications, such as in artificial infant milk. Although extensive research has been conducted on the LF’s benefits, little focus has been placed on evaluating the stability of native LF under enzymatic digestion or thermal denaturation conditions during both industrial processing and domestic preparation. This oversight necessitates precise determinations considering, as initially discussed, that LF is sensitive to environmental conditions, including heat, pH, co-present proteins or minerals, and oxidation. It can undergo structural changes and lose its bioactivity when exposed to unfavourable conditions in the organism and during storage or processing [176]. For example, in a compelling study, researchers developed a kinetic model to assess the thermal denaturation of native LF in two samples of raw whole cow’s milk with different iron saturation levels (11.6% and 21.9%). The temperatures tested ranged from 65 °C to 95 °C for varying holding times from 2 s to 300 s to mimic HTST and UHT pasteurisation conditions. The heat stability of LF in the milk with lower iron saturation levels was lower until heating to 85 °C, where the heat stabilities became similar. At 95 °C, the denaturation rates of LF in milk with higher iron saturation levels were twice as high as those in milk with lower iron saturation levels. Heating to 121 °C denatured all LF in both milk samples within 4 s [176]. Additionally, it has been reported that a protein’s glycosylation patterns may influence its thermal stability [177]. Specifically, in the case of LF, glycosylation at Asn281 shields bovine lactoferrin from trypsin cleavage at Lys282, whereas the glycosylation of human lactoferrin does not offer similar protection [178]. The in vivo digestion of LF remains poorly understood and is characterised by a range of conflicting assertions. While some studies report that rh LF is completely degraded in the upper gastrointestinal tract, others indicate that over 60% of the orally administered bovine LF remains structurally intact through the gastric stage [179,180]. Factors such as gastric emptying rate and the buffering capacity of food significantly influence LF’s in vivo digestion rate [175]. The digestion dynamics of LF in human infants and newborns are less disputed, largely due to their immature digestive systems, characterised by higher intragastric pH and gastric emptying rates compared to adults, preventing complete LF digestion. This is supported by detecting undigested LF in babies’ faecal extracts [181,182]. However, no in vivo study definitively maps the extent and nature of LF digestion at different ages. Parameters including the types of enzymes, their activities, enzyme–substrate ratios, time, and pH should be aligned with the individual’s age, fasting/feeding state, and other physicochemical conditions within the human digestive system. While oral administration is the most common method for delivering LF into the human body, it presents challenges that must be overcome to maximise its benefits. The current literature suggests that LF does not remain structurally intact through the gastric stage of digestion, indicating the need for protective measures. Microencapsulation and PEGylation are the most effective techniques for transporting LF to intestinal absorption sites [175]. It is important to note that ongoing research and technological advancements may address some of these limitations. For example, developing LF analogues and novel delivery systems may help overcome bioavailability, stability, and production issues and enhance specific properties. For instance, Tomita and colleagues’ 1991 research, which involved enzymatically digesting bovine LF with pepsin, identified a cleavage peptide later termed bovine lactoferricin [183,184]. This peptide demonstrated enhanced antibacterial properties compared to the original LF forms. Following this discovery, various peptides have been either generated through the enzymatic digestion of LFs or synthetically produced [56]. Additionally, the analogues of LF can be synthesised or engineered to replicate particular functional domains or regions of LF that are key to its biological effects. The emphasis is on these active areas to develop synthetic peptides or compounds capable of selectively influencing immune reactions, hindering microbial proliferation, or achieving other targeted outcomes. Examples of such developments include Lactoferrampin and Lactoferrin-Chimera [56]. Research on LF analogues is a burgeoning field with potential uses beyond treating allergic conditions. However, it is important to recognise that these LF analogues’ clinical effectiveness and safety are under investigation. More studies must comprehensively assess their benefits and identify any possible adverse effects.

**Figure 4:**
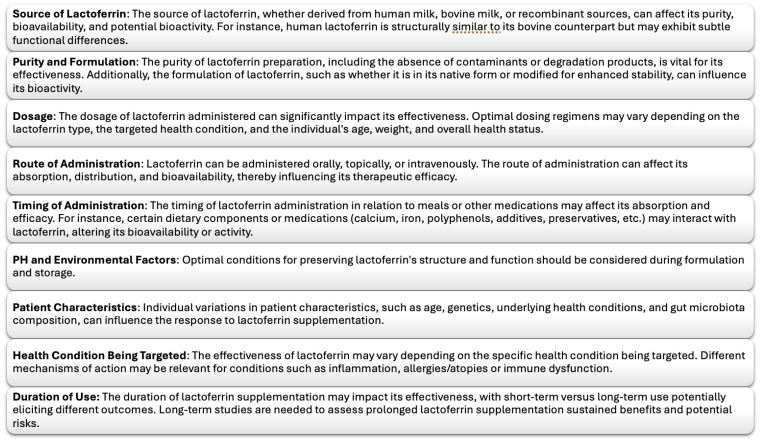
Key factors influencing the efficacy of LF. Considering these factors is essential for maximising lactoferrin’s therapeutic potential in various clinical and nutritional applications. Further research is needed to elucidate the optimal strategies for harnessing its benefits effectively.

From its antimicrobial properties to its immune-modulating effects, lactoferrin is extensively studied for its therapeutic potential. However, the efficacy of lactoferrin can be influenced by a myriad of factors, ranging from its source to its delivery method. Understanding these factors is crucial for optimising its effectiveness in various applications or designing randomised studies. Several key factors are summarised in Figure 4. In addition, despite the extensive research on LF supplementation and its health benefits, there needs to be more scientific research regarding studies evaluating the impact of LF-enriched milk formula, milk-inclusive diet, or LF supplementation on systemic and local lactoferrin levels. Most studies focus primarily on the disease outcomes related to supplementation, leaving a critical need for investigations that address how these interventions affect lactoferrin distribution and concentration in various body compartments, particularly the respiratory system. Another essential aspect that we should emphasise relates to analytical strategies for LF detection in biological fluids: LF, even more, has been recognised as a biomarker for various diseases, necessitating the development of accurate, cost-effective, rapid, and standardised detection methods. An interesting review examines several analytical techniques, including immunoassays, instrumental analysis, and sensor-based methods, highlighting their advantages and limitations [185]. For instance, among immunoassays, Enzyme-Linked Immunosorbent Assay (ELISA) offers high accuracy and selectivity and is capable of detecting LF in various samples like the serum, saliva, tears, and milk products despite it being labour-intensive and expensive; radial immunodiffusion (RID) is a simple, quantitative approach but time-consuming and lacks precision. In comparison, instrumental methods such as UV-Vis spectroscopy and High-Performance Liquid Chromatography (HPLC) are employed for LF quantification. UV-Vis spectroscopy is suitable for pure samples but struggles with impurities. Reversed Phase-HPLC (RP-HPLC) has shown high sensitivity and accuracy; however, these methods require extensive sample preparation and are limited by the presence of proteins with similar properties. In contrast, sensor-based methods, though promising, face challenges with environmental factors and reproducibility. Fluorescence-based biosensors, such as microfluidic paper-based analytical devices (μPAD), provide visual, low-cost, and high-sensitivity detection. Electrochemical biosensors convert biological interactions into electrical signals, offering high accuracy and low detection limits. Surface Plasmon Resonance (SPR) sensors enable real-time analysis with high accuracy but are expensive and instrument-dependent [185]. Future advancements should focus on creating a quick, cost-effective, and reliable LF detection system, integrating the strengths of current techniques while mitigating their drawbacks.

## 9. Conclusions

Current allergy treatments, including allergen avoidance, antihistamines, and corticosteroids, primarily offer symptomatic relief and are often limited in effectiveness. On the other hand, allergen immunotherapy, the only therapy that modifies the disease’s natural history inducing immune tolerance, faces challenges due to duration, limited patient adherence, and high expenses. Consequently, there is a continuous and active pursuit of novel therapeutic approaches. Lately, there has been a growing interest in food-derived compounds based on the widespread belief that natural substances are inherently healthy and conducive to balance. This perception positions them as attractive options for supplementary treatment in cases of immune dysregulation. While further studies are necessary, LF may be a promising, safe, and natural adjunctive treatment with great appeal, especially in paediatrics. Its potential utility in preventing ongoing prophylaxis and treating allergic disorders is highlighted by its anti-inflammatory, immunomodulatory, and antioxidant properties.

While lactoferrin has demonstrated potential in numerous studies, more substantial clinical proof is still required to validate its effectiveness in addressing certain diseases or health conditions. More in-depth research, encompassing rigorously structured clinical trials, is needed to ascertain the most effective dosage, method of administration, and specific therapeutic uses for LF in children with allergic diseases. Some individuals might exhibit allergic responses to LF in isolated instances, especially those with pre-existing milk protein allergies.

We hope this review has the potential to stimulate clinical study that can help address a research gap or unanswered questions in this field, providing evidence, scientific validation, and evidence-based support for the effectiveness and safety of LF-based intervention in allergic diseases. The results of well-conducted clinical trials will contribute to the body of scientific literature and help guide clinical practice and decision making in developing personalised approaches to manage and improve the lives of individuals affected by allergic disorders. As LF continues to capture the attention of researchers, its multifaceted properties and diverse applications make it an intriguing subject of study with a rich history and a promising future.

## Figures and Tables

**Table 1:** Characteristics and results of the studies on the effect of various forms of LF applied alone or in combination with other compounds on AR. AAR: Active Anterior Rhinomanometry; Dpt: *Dermatophagoides pteronyssinus*; MCT: Mucociliary Transport Time; NPT: nasal provocation test; VAS: Visual Analogue scale.

Reference	First Author	Publication Year	Study Model	LF Type	Administration Route	Effects
[130]	Passali D	2015	Human, in vivo	Associated with carboximetil b-glucan, D-panthenol, and dipotassium glycyrrhizinate.	Intranasal spray. Two puffs into each nostril two times a day over the course of 4 weeks	The improvement of AR symptoms evaluated with VAS (nasal obstruction, sneezing, watery eyes, rhinorrhea, and overall symptom burden). The improvement of AAR and MCTt.
[118]	Wang SB	2013	Murine, in vivo	Recombinant human	Intranasal instillation 100 μg LF	Decrease in eosinophils, goblet cells, and granulocytes. The downregulation of Th2-related cytokines and transcription factors (IL-5 and GATA-3), Th17-related cytokines and transcription factors (IL-17 and ROR-c).
[129]	Passali D	2012	Human, in vivo	Associated with carboximetil b-glucan, D-panthenol, and dipotassium glycyrrhizinate.	Intranasal spray. Two puffs into each nostril two times a day over the course of 4 weeks	The improvement of AR symptoms evaluated with VAS (nasal obstruction, sneezing, watery eyes, rhinorrhea, and overall symptom burden).
[65]	Choi GS	2010	Human, in vivo	Endogenous	Nasal lavage fluid serum	LF expression was upregulated after NPT. Serum LF level is associated with the phenotype of Dpt-sensitive AR. Serum LF level in combination with the serum Dpt-specific IgE level, may be a marker for early detection of AR.

**Table 2:** Characteristics and results of the studies on the effect of various forms of LF applied alone or in combination with other compounds on Asthma. AHR: airway hyperresponsiveness. BALF: bronchoalveolar lavage fluid. DCs: dendritic cells. OVA: Ovalbumin. ROS: reactive oxygen species. RWE: ragweed extract.

References	First Author	Publication Year	Study Model	LF Type	Administration Route	Effects
[138]	Lin CC	2022	Murine, in vitro and in vivo	Unspecified	Oral administration 100 mg/kg or 300 mg/kg	The improvement of OVA-induced AHR and pulmonary inflammation and suppression of the production of OVA-induced Th2 Cytokines. Increase in anti-inflammatory cytokines in the BALF regulation of OVA-specific IgG1 and IgE secretion in the serum. Decrease in OVA-specific Th2 responses in the spleen. The downregulation of the surface molecules CD80 and CD86 in DCs in the spleen of OVA-treated mice. Decrease in the capacity of DCs to stimulate OVA-specific Th2-cell responses in vitro.
[144]	Dasgupta A	2021	Human, in vivo	Endogenous	Sputum	LF biomarkers for frequent exacerbators.
[142]	Shinagawa K	2020	Human, in vivo	Bovine	Inhalation	Occupational asthma.
[145]	Haghi M	2019	Human, in vitro	Peptide derived from the N-terminal domain of human lactoferrin	Transwell passage	Cell-penetrating peptide for delivery therapeutics across respiratory epithelia.
[144]	Nagaoka K	2017	Murine, in vivo	Human	Intranasal inoculation	The induction of airway inflammation: increased AHR, eosinophils in BALF, serum LF-specific IgG levels, and mRNA levels of IL-13, eotaxin 1, and eotaxin 2.
[141]	Bournazou I	2010	Human, in vitro	Human milk-derived and neutrophil-derived	Added to the upper chamber along with eosinophils	The control of eosinophil infiltration in atopic inflammatory conditions.
[140]	Chodaczek G	2007	Murine, in vivo	Human milk-derived. Apo-LF (100 μg) and holo.LF (100 μg)	Intranasal	Diminishing the effect of oxidative stress in allergic airway inflammation.
[139]	Kruzel ML	2006	Human, in vitro; murine, in vivo	Human milk-derived. Apo-LF (100 μg) and holo.LF (100 μg)	Intranasal	Lowering RWE-induced increase in ROS levels in bronchial epithelial cells.

## Supplementary Materials

The following supporting information can be downloaded at: https://www.mdpi.com/article/10.3390/nu16121906/s1, Figure S1: Flow diagram for the review process (Allergic rhinitis); Figure S2: Lactoferrin Clinical Application (Asthma).
