# Supplementary material for: Exploring the Role of Lactoferrin in Managing Allergic Airway Diseases among Children: Unrevealing a Potential Breakthrough
Source: Nutrients. 2024 Jun 17;16(12):1906. doi: 10.3390/nu16121906 (PMC11206375; doi:10.3390/nu16121906)
Supplement: Supplementary file 1 [file nutrients-16-01906-s001.zip › nutrients-3016640-supplementary.pdf]

nutrients

Supplementary Materials

Review

Exploring the Role of Lactoferrin in Managing Allergic Airways Diseases Among Children:Unrevealing a Potential Breakthrough

Alessandra Gori [1], Giulia Brindisi [1], Maria Daglia [2,3], Michele Miraglia del Giudice [4], Giulio Dinardo [4], Alessandro Di Minno [2,5], Lorenzo Drago [6,7], Cristiana Indolfi [4], Matteo Naso [8], Chiara Trincianti [8], Enrico Tondina [9], Francesco Paolo Brunese [10], Hammad Ullah [2], Attilio Varricchio [11], Giorgio Ciprandi [12] and Anna Maria Zicari [1,*] on behalf of the Nutraceutical and Medical Device Task Force of the Italian Society of Pediatric Allergy, Immunology (SIAIP)

[1] Department of Mother-Child, Urological Science, Sapienza University of Rome, 00161 Rome, Italy.
[2] Department of Pharmacy, University of Napoli Federico II, Via D. Montesano 49, 80131 Naples, Italy
[3] International Research Center for Food Nutrition and Safety, Jiangsu University, Zhenjiang 2013, China
[4] Department of Woman, Child and General and Specialized Surgery, University of Campania "Luigi Vanvitelli", Naples, Italy
[5] CEINGE-Biotecnologie Avanzate, Via Gaetano Salvatore 486, 80145 Naples, Italy
[6] Laboratory of Clinical Microbiology & Microbiome, Department of Biomedical Sciences for Health, University of Milan, Italy
[7] UOC Laboratory of Clinical Medicine, MultiLab Department, IRCCS Multimedica, Milan, Italy.
[8] Allergy Center, IRCCS Istituto Giannina Gaslini, Genoa, Italy
[9] Pediatric Clinic, Fondazione IRCCS Policlinico San Matteo, Pavia, Italy
[10] Primary Care Paediatrics, ASL Caserta, Caserta, Italy
[11] Department of Otolaryngology, University of Molise, 86100 Campobasso, Italy
[12] Allergy Clinic, Casa di Cura Villa Montallegro, Genoa, Italy
* Correspondence: Anna Maria Zicari[1], annamaria.zicari@uniroma1.it

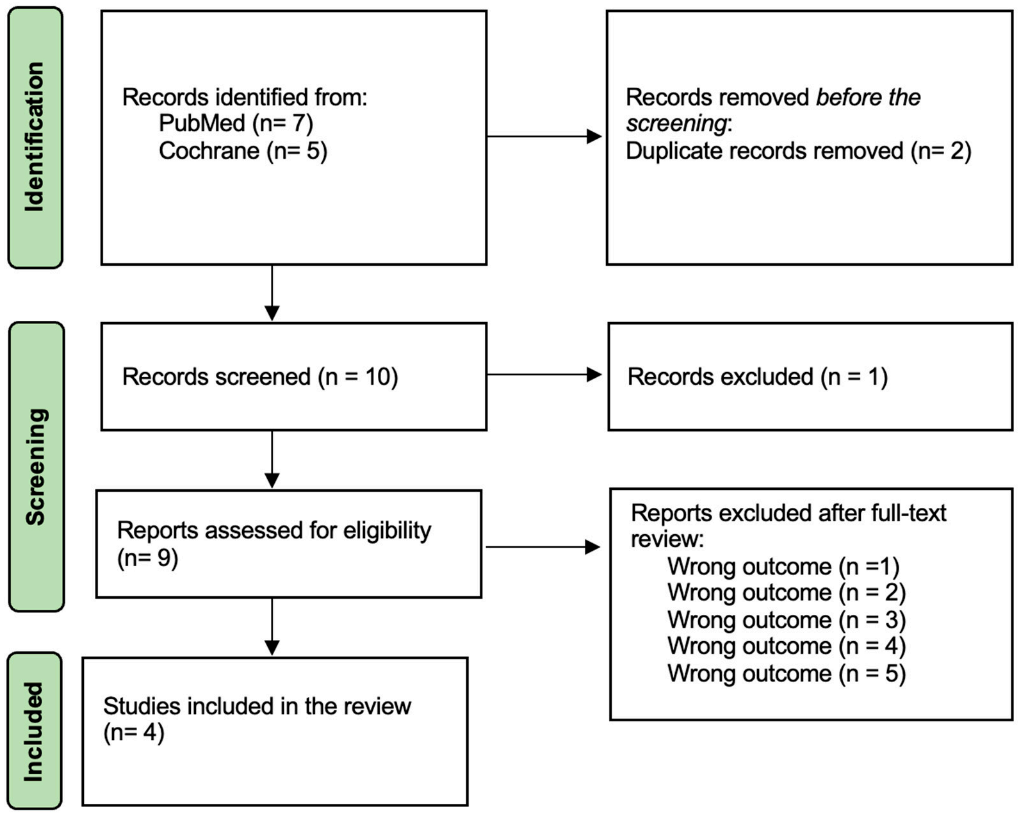

Figure S1. Flow diagram for the review process.

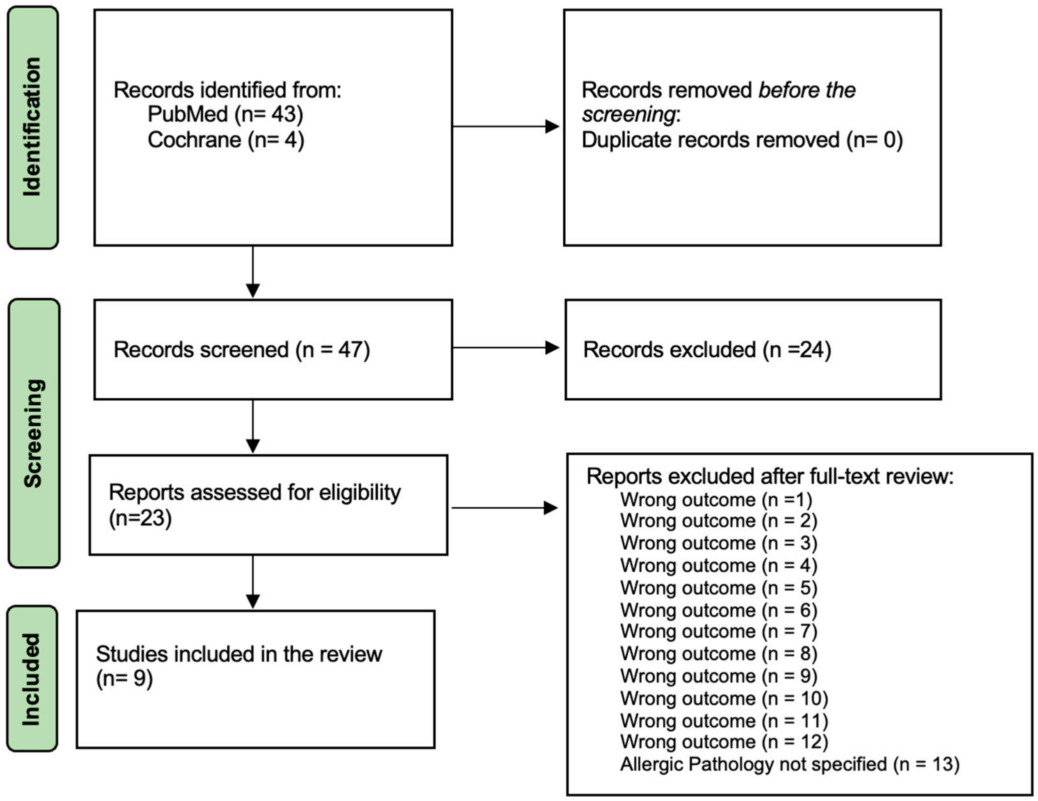

Figure S2. Flow diagram for the review process.
